# *Syringa oblata Lindl*. Aqueous Extract Is a Potential Biofilm Inhibitor in *S. suis*

**DOI:** 10.3389/fphar.2017.00026

**Published:** 2017-01-30

**Authors:** Jingwen Bai, Yanbei Yang, Shuai Wang, Lingfei Gao, Jianqing Chen, Yongzhi Ren, Wenya Ding, Ishfaq Muhammad, Yanhua Li

**Affiliations:** College of Veterinary Medicine, Northeast Agricultural UniversityHarbin, China

**Keywords:** proteomics, *Streptococcus suis*, *Syringa oblata Lindl.*, biofilm formation

## Abstract

*Streptococcus suis* (*S. suis*) is a zoonotic pathogen that causes severe disease symptoms in pigs and humans. *Syringa oblata Lindl.* distributed in the middle latitudes of Eurasia and North America were proved as the most development potential of Chinese Medicine. In this study, biofilm formation by *S. suis* decreased after growth with 1/2 MIC, 1/4 MIC, or 1/8 MIC of *Syringa oblata Lindl.* aqueous extract and rutin. Scanning electron microscopy analysis revealed the potential effect of *Syringa oblata Lindl.* aqueous extract and rutin against biofilm formation by *S. suis*. Using iTRAQ technology, comparative proteomic analyses was performed at two conditions: 1/2 MIC of *Syringa oblata Lindl.* aqueous extract treated and non-treated cells. The results revealed the existence of 28 proteins of varying amounts. We found that the majority of the proteins were related to cell growth and metabolism. We also found that *Syringa oblata Lindl.* Aqueous extract affected the synthesis enzymes. In summary, *Syringa oblata Lindl.* aqueous extract might be used to inhibit the biofilm formation effectively by *S. suis*, and the active ingredients of the *Syringa oblate Lindl.* aqueous extract is rutin. The content of rutin is 9.9 ± 0.089 mg/g dry weight.

## Introduction

*Streptococcus suis* (*S. suis*) is a major bacterial pathogen of young pigs and a worldwide economic problem for the pig industry (Goyette-Desjardins et al., 2014). Furthermore, *S. suis* is emerging as a zoonotic pathogen associated with meningitis and septicaemia in humans (Ferrando et al., 2014). In one often-cited statistic sourced from the US National Institutes of Health (NIH), biofilms are indicated as the cause of >60% of all clinical microbial infections (Abbanat et al., 2014). Moreover, some studies have demonstrated that *S. suis* has the ability to form biofilms (Brown et al., 1988; Brady et al., 2008). Biofilms are defined as consortia of microorganisms that are attached to a biotic or abiotic surface. In addition, microorganisms embedded in the biofilm offers more resistant to desiccation, environmental stress (nutritional or oxidative stress), and UV lightexposure (Lee et al., 2008; Rao et al., 2008).

The *S. suis* serotypes are determined by the antigenicity of capsule. To date, *S. suis* have 33 serotypes according to antigenic differences in the capsular polysaccharide (CPS) (Wang et al., 2011). The cps genes code for the production of capsule (Smith et al., 1999). *S. suis* serotyping based on variation in the capsule structures encoded by cps locus genes accountable for capsule formation (Liu et al., 2013).

Now days, the majority of drugs produced from naturally occurring molecules, especially the anti-infective agents. Newman and Cragg surveyed that natural products, or drugs obtained from natural product scaffolds, account for >75% of the 97 accepted antibacterial New Chemical Entities intercalate over the period 1981–2006, shows the significance of natural products used in clinics against infectious diseases (Taylor, 2013). The discovery of new drugs for curing various diseases mostly comes from biologically active natural products, especially the plant-derived ones (Coleman et al., 2010; Koh et al., 2013). Particularly, some plants grow in an environment that has high bacterial density and so they have co-existed with bacteria during their evolution. Accordingly, the plants may have evolved protective mechanisms against bacterial infections, and may even produce bacterial biofilm inhibitors (Zhu et al., 1998; Ding et al., 2011). *Folium syringae* leaves are mainly from the dry leaves of *Oleaceae* plant: *Syringa oblate Lindl., Syringa diatata Nakai* and *Syringa vulgaris* L., which were proved as the most development potential of Chinese Medicine through its plant resources, chemical constituents, pharmacological action and clinical application. *Syringa* has been widely cultivated in the northern parts of China and Korea. The traditional Chinese treatment uses the leaf, flowerbud, and bark of *Syringa* to treat various infections, heal inflammations, dampness and acute icteric hepatitis (Feng et al., 2009). In Korea, the stem bark of *Syringa* has been used for the treatment of tooth pain, intestinal disorders and diarrhea (Park et al., 1999).

We have analyzed the relationship between the spectrum and the impact of *Syringa oblata Lindl.* aqueous extract on *S. suis* biofilms *in vitro*. According to HPLC fingerprint and anti-biofilm activity test, gray relational analysis was applied to find the active compositions. According to the relational grade, rutin make significant contribution to anti-biofilm activity. Rutin is a well-known and widely used citrus flavonoid glycoside between flavonol quercetin and disaccharide rutinose. It is found in many foods, such as buckwheat, onion, lemon, apple, orange, and grapefruit. Rutin has a lot of benefit pharmacological effects, such as anti-inflammatory, antimicrobial, antioxidant, and antihypertensive effects (Erlund et al., 2000; Middleton et al., 2000).

Our laboratory recently reported that sub-MICs of emodin and sub-MICs of erythromycin inhibited biofilm formation by *S. suis* ATCC 700794 (Yang et al., 2015; Zhao et al., 2015). However, the relationship between *Syringa oblata Lindl.* and biofilm formation by *S. suis* remains poorly understood. The objective of this study was to search new potential inhibitors for the control of biofilm formation by *S. suis*; and to describe the use of proteomics to better understand the impact of *Syringa oblata Lindl.* aqueous extract on *S. suis* biofilms *in vitro*.

## Materials and Methods

### Preparation of *Syringa oblata Lindl.* Aqueous Extract

*Syringa oblata Lindl.* was collected in Northeast Agricultural University (Harbin, Heilongjiang, China). Taxonomic identi fication was confirmed by Professor Xiuju Wu (Heilongjiang University of traditional Chinese Medicine, Harbin, China). A coarsely powdered, air-dried, *Syringa oblata Lindl.* (leaves, 200 g) was boiled in 2 L of distilled water for 45 min, decanted and filtered. The filtrate was evaporated to dryness in an oven set at 60°C. The dried *Syringa oblata Lindl.* extract was weighed and reconstituted to a concentration of 100 mg/mL.

### HPLC and HPLC-ESI-MS Analysis of *Syringa oblata Lindl.* Aqueous Extract

On-line HPLC measurements were performed on Waters Alliance HPLC system (Shimadzu Corporation, Kyoto, Japan) consisting of a binary pump and a UV/V is detector. The separation was achieved using a DL-Cl8 column (4.6 mm × 250 mm, 5 μm, Japan) at 25°C. The mobile phase was composed of acetonitrile (A) and 0.1% formic acid (B). The rate of flow was 1.0 mL/min. The volume injected was 20 μL and the detection wavelength was 245 nm.

For LC–MS analysis, the Agilent 3000 HPLC system was coupled on-line to an LC/MSD Trap SL Plus spectrometer (Agilent, Corp., Massachusetts, American) equipped with electrospray ionization (ESI) source. In order to ionize the target compound, ESI in both positive- and negative-ion modes was investigated. The MS was operated in MRM mode, and the capillary voltage and the ion spray voltage were set at 3.0 and 5.5 kV, respectively. Ultrahigh-purity helium was used as the collision gas (10 p.s.i.) and high-purity nitrogen as the nebulizing gas (12 p.s.i.). The source temperature was set at 300°C. For full scan MS analysis, the spectra were recorded in the range m/z 100–1000.

### Identification and Quantification of Rutin

Rutin in the *Syringa oblata Lindl*. aqueous extract was identified by comparing its mass spectrum with literature data (Regos et al., 2009; Zhao et al., 2013), and comparing the HPLC retention time with that of the authentic standard. Quantification of rutin in the aqueous extract was carried out based on linear calibration plot of the peak area in HPLC at 245 nm against concentration using the external standard method.

### Experimental Bacterial Strain and Culture Conditions

*Streptococcus suis* ATCC 700794 was used in this study. Bacteria were cultured at 37°C in Todd-Hewitt broth (THB; Summus, Ltd, Harbin, Heilongjiang, China) or Todd-Hewitt broth agar (THA) added with 5% (v/v) fetal bovine serum (Sijiqing, Ltd, Hangzhou, Zhejiang, China). In order to obtain THA, The original THB was added with 1.8% Bacto-agar (Difco Laboratories). The media were sterilized in autoclave (30 min at 115°C) and before inoculation, all strains were transferred from the stock cultures to THA and incubated aerobically at 37°C for 16 h. Then, all strains were sub-cultured one more time under the same conditions. The cultures were used for the minimal inhibitory concentration (MIC) assays and the biofilm assays.

### Determination of MIC

Minimal inhibitory concentration of *Syringa oblata Lindl.* aqueous extract and rutin (dissolved in Methanol) (Guoyao, Ltd, China) was determined three times using the protocol described by Wiegand et al. (2008), with a few modifications. Briefly, The sterile saline solutions were used for the dilution of overnight cultures of *S. suis* to obtain the turbidity of a so-called McFarland 0.5 standard (corresponding to 1 × 10^8^ colony-forming units [CFUs]/mL). Then, 5% (v/v) fetal bovine serum was added to sterile THB to dilute the cultures of *S. suis* (1:100). 100 μL of each sample was added to the wells of a Costar^®^3599 96-well plate (Corning, NY, USA) containing serial dilutions of *Syringa oblata Lindl.* aqueous extract and rutin in culture medium (100 μL). Bacteria grown as control were cultured in the absence of *Syringa oblata Lindl.* aqueous extract and rutin. After incubation for 24 h at 37°C, the MIC was determined as the lowest concentration of *Syringa oblata Lindl.* aqueous extract and rutin that completely inhibited *S. suis* growth.

### Biofilm Assay

*Streptococcus suis* strain was cultured overnight and diluted to an optical density of 0.1 at 660 nm (OD 660). Same amount of *S. suis* was grown in a Costar^®^ 3599 96-well plate in the presence of 1/2 MIC, 1/4 MIC, 1/8 MIC, or 1/16 MIC of *Syringa oblata Lindl.* aqueous extract and rutin. *S. suis* ATCC 700794 treated without *Syringa oblata Lindl.* aqueous extract and rutin was served as a control. Biofilms were treated as described by Yang et al. (2015) with some modifications. Briefly, the medium, free-floating bacteria, and loosely bound biofilm were removed by aspiration, and the wells were washed thrice with sterile physiological saline. The remaining bacteria were fixed with 200 μL methanol (99%) (Guoyao, Ltd, China) per well, and after 15 min, plates were dried and emptied. Then, plates were stained for 5 min with 200 μL crystal violet (2%) (Guoyao, Ltd, China) per well. The excess stain was washed with water. Then the dye was resolubilized with 200 μL glacial acetic acid (33%) (Guoyao, Ltd, China) per well. A microplate reader (DG5033A, Huadong, Ltd, Nanjing, Jiangsu, China) was used to quantify the amount of released stain at absorbance of 570 nm.

### Scanning Electron Microscopy (SEM)

Scanning electron microscope (SEM) was used to examine the *S. suis* biofilm (refer to Bedran et al., 2014). Briefly, 1 mL of *S. suis* were diluted to an optical density of 0.1 at 600 nm (OD_600_) in culture without *Syringa oblata Lindl.* aqueous extract or with 1/2 MIC of *Syringa oblata Lindl.* aqueous extract was added into wells of a 6-well plate containing glass slide. The same condition was treated for rutin. After 24 h incubation, medium and free-floating bacteria were removed. The biofilms were incubated overnight in fixation buffer (4% (w/v) paraformaldehyde, 2.5% (w/v) glutaraldehyde, 2 mM CaCl_2_ in 0.2 M cacodylate buffer, pH 7.2), washed with 0.1 M cacodylate buffer pH 7.0 (3 min × 20 min) and post-fixed for 90 min at room temperature in 1% (w/v) osmic acid containing 2 mM potassium ferrocyanide and 6% (w/v) sucrose in cacodylate buffer. The samples were dried, gold sputtered with an ion sputtering instrument (current 15 mA, 2 min) and finally observed using SEM (FEI Quanta, Netherlands).

### Protein Digestion and iTRAQ Labeling

Protein was extracted from *S. suis* cells at two different conditions (1/2 MIC of *Syringa oblata Lindl.* aqueous extract treated cells and non-treated cells). iTRAQ analysis was implemented at Shanghai Applied Protein Technology, Co. Ltd (APT, Shanghai, China). Three biological replicates were evaluated to minimize the influence of less reliable quantitative information.

Protein digestion was carried out according to the reported FASP procedure (Wisniewski et al., 2009). Briefly, 200 μg of proteins for each sample were incorporated into 30 μL STD buffer (4% SDS, 100 mM DTT, 150 mM Tris-HCl pH 8.0). The detergent, DTT and other low-molecular-weight components were removed using UA buffer (8 M Urea, 150 mM Tris-HCl pH 8.0) by repeated ultrafiltration (Microcon units, 30 kD). Then 100 μL 0.05 M iodoacetamide in UA buffer was added to block reduced cysteine residues and the samples were incubated for 20 min in the dark. The filters were washed three times with 100 μL UA buffer and two times with 100 μL DS buffer (50 mM triethylammoniumbicarbonate at pH 8.5) respectively. Then, the protein suspensions were digested with 2 μg trypsin (Promega) in 40 μL DS buffer overnight at 37°C, and the filtrate (peptides) were collected. The peptide content was quantified by UV light spectral density at 280 nm using an extinctions coefficient of 1.1 of 0.1% (g/l) solution that was calculated on the basis of the frequency of tryptophan and tyrosine in vertebrate proteins.

For the iTRAQ labeling, the peptides were labeled with the 8-plex iTRAQ reagent by following the manufacturer’s instructions (Applied Biosystems). Each iTRAQ reagent was dissolved in 70 μL of ethanol and added to the respective peptide mixture. The peptides from the *S. suis* biofilms treated by Sy*ringa oblata Lindl.* aqueous extract were labeled with 115 isobaric reagent, and the peptides from the non-treated *S. suis* biofilms were labeled with 116 isobaric reagent. Then, the samples were multiplexed and vacuum dried. Three independent biological experiments were performed.

### Peptide Fractionation with Strong Cation Exchange (SCX) Chromatography

Strong cation exchange (SCX) chromatography AKTA Purifier system (GE Healthcare) was used for iTRAQ labeled peptides fractionation. The dried peptide mixture was reconstituted and acidified with 2 mL buffer A (10 mM KH_2_PO_4_ in 25% of ACN, pH 2.7) and loaded onto a PolySULFOETHYL 4.6 mm × 100 mm column (5 μm, 200 Å, PolyLC, Inc., Columbia, MD, USA). The peptides were eluted at a flow rate of 1 ml/min with a gradient of 0–10% buffer B (500 mM KCl, 10 mM KH_2_PO_4_ in 25% of ACN, pH 2.7) for 2 min, 10–20% buffer B for 25 min, 20–45% buffer B for 5 min, and 50–100% buffer B for 5 min. The separation was monitored by absorbance at 214 nm, and the fractions were collected after every 1 min. The obtained fractions (approximately 30 fractions) were combined into 10 pools and desalted on C18 Cartridges [Empore^TM^ SPE Cartridges C18 (standard density), bed I.D. 7 mm, volume 3 ml, Sigma]. All fractions were concentrated by vacuum centrifugation and reconstituted in 40 μL of 0.1% (v/v) trifluoroacetic acid. And samples were stored at -80°C until LC–MS/MS analysis.

### Liquid Chromatography (LC) – Electrospray Ionization (ESI) Tandem MS (MS/MS) Analysis by Q Exactive

Q Exactive mass spectrometer was used for experiments that were coupled to Easy nLC (Proxeon Biosystems, now Thermo Fisher Scientific). Each fraction (10 μL) was injected for the nanoLC–MS/MS analysis. The peptide mixture (5 μg) was loaded onto a C18-reversed phase column (Thermo Scientific Easy Column, 10 cm long, 75 μm inner diameter, 3 μm resin) in buffer A (0.1% formic acid) and separated with a linear gradient of buffer B (80% acetonitrile and 0.1% formic acid) at a flow rate of 250 nl/min controlled by IntelliFlow technology over 140 min. MS data were acquired using a data-dependent top10 method dynamically choosing the most abundant precursor ions from the survey scan (300–1800 m/z) for HCD fragmentation. Determination of the target value is based on predictive Automatic Gain Control (pAGC). Dynamic exclusion duration was 60 s. Survey scans were acquired at a resolution of 70,000 at m/z 200, and resolution for HCD spectra was fixed at 17,500 at m/z 200. 30 eV was normalized collision energy and the underfill ratio, which shows the minimum percentage of the target value likely to be reached at maximum fill time, was defined as 0.1%. The experiment run with the peptide recognition mode enabled.

### Sequence Database Searching and Data Analysis

The MS/MS spectra were searched using the MASCOT engine (Matrix Science, London, UK; version 2.2) in the Proteome Discoverer 1.3 (Thermo Electron, San Jose, CA, USA) against Uniprot *Streptococcus suis*. fasta database (uniprot *streptococcus suis*. fasta, 38369 sequences, downloaded at November 4th, 2013) and the decoy database. The following options were used for protein identification. MS/MS tolerance = 0.1 Da, Peptide mass tolerance = 20 ppm, Enzyme = Trypsin, Missed cleavage = 2, Fixed modification: Carbamidomethyl (C), iTRAQ 8plex (K), iTRAQ 8plex (N-term), Variable modification:Oxidation (M), FDR ≤ 0.01.

### Statistical Analysis

Assays were run in triplicate and the mean ± standard deviation were calculated. Data were analyzed using the Student’s *t*-test.

## Results and Discussion

### Identification and Quantification of Rutin in *Syringa oblata Lindl.* Aqueous Extract

**FIGURE 1:**
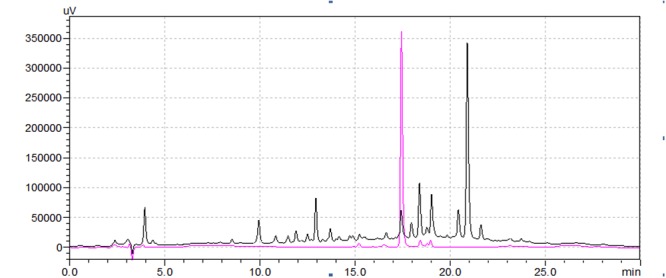
**The HPLC chromatograms of the *Syringa oblata Lindl*. aqueous extract (1) and the rutin standard (2)**.

**FIGURE 2:**
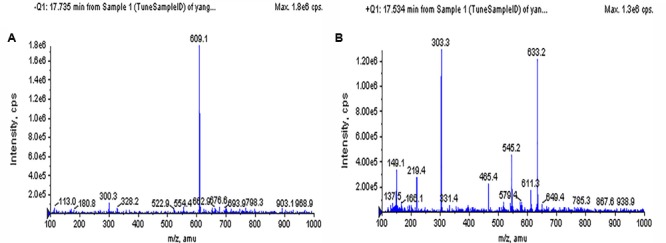
**Mass spectra of *Syringa oblata Lindl.* aqueous extract. (A)** MS spectra in negative mode, **(B)** MS spectra in positive mode.

The chromatographic characteristics of the compound such as retention time and molecular ions were established under the experimental conditions to determine the rutin in *Syringa oblata Lindl.* aqueous extract. The typical HPLC chromatograms of sample solution and standard rutin solution were shown in **Figure 1**. As could be seen, the retention time of the rutin in sample was in good agreement to the authentic compound (17.66 min). In addition, the sample was also analyzed via ESI-MS in the negative and positive ion modes (**Figure 2**). Note that the ESI (-) – MS data (**Figure 2A**), it demonstrated a molecularion ([M – H]^+^) at m/z = 609.2 and a fragment ion at m/z = 299.8. The fragment ion at m/z 299.8 was produced leaving the rutinoside, suggesting that rutin was converted to quercetin (Regos et al., 2009; Zhao et al., 2013). The sample analysis via ESI-MS in the positive ion mode was shown in **Figure 2B**, molecular cation was detected mainly in [M + Na]^+^ form (m/z 633.2), and minor [M + H]^+^ (m/z 611.3) ion was also detected. The spectrum showed abundant ions at m/z 303.3 (loss of rutinoside) (Regos et al., 2009; Zhao et al., 2013). Thus, all of these evidences confirmed the existence of rutin in *Syringa oblata Lindl.* aqueous extract. Quantification of rutin was performed based on the calibration curve which was obtained by plotting peak areas vs. six different concentrations of the standard solutions (0.0126, 0.0252, 0.0504, 0.1008, 0.0152, and 0.2016 mg/mL). The calibration curve equation was y = 3E+07x+93411, *R*^2^ = 0.9991. As a result, the mean value of rutin in the aqueous extract of *Syringa oblata Lindl*. was found at 9.9 ± 0.089 mg/g dry weight, with a recovery yield of 101.43 ± 0.99%.

### Effect of *Syringa oblata Lindl.* Aqueous Extract and Rutin against Biofilm Formation *In vitro*

**FIGURE 3:**
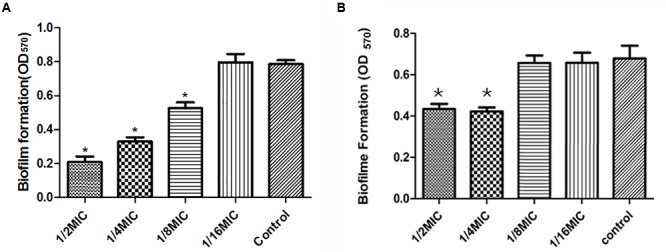
**(A)** Effect of sub-MICs of *Syringa oblata Lindl.* aqueous extract on biofilm formation by *Streptococcus suis* ATCC700794. **(B)** Effect of sub-MICs of rutin on biofilm formation by *S. suis* ATCC700794. Data are expressed as means ± standard deviations. Controls refer to the absence of *Syringa oblata Lindl.* aqueous extract. Significant decrease (^∗^*p* < 0.05) compared to control bacteria grown in the absence of *Syringa oblata Lindl.* aqueous extract.

Minimal inhibitory concentration of *Syringa oblata Lindl.* aqueous extract against *S. suis* ATCC 700794 was determined as 50 mg/mL. Thereafter, we evaluated the action of *Syringa oblata Lindl.* aqueous extract on biofilm growth *in vitro*. As reported in **Figure 3A**, when the culture medium was added with 1/2 MIC, 1/4 MIC, and 1/8 MIC of *Syringa oblata Lindl.* aqueous extract, the biofilms by *S. suis* were reduced (*p* < 0.05). There was no pronounced effect for 1/16 MIC of *Syringa oblata Lindl.* aqueous extract on biofilm formation of *S. suis* (*p* > 0.05). We also evaluated the action of rutin on biofilm growth *in vitro*. The MIC against *S. suis* was 0.3125 mg mL^-1^. Rutin at 1/2 MIC and 1/4 MIC caused a significantly higher reduction in the biofilm-forming ability of *S. suis* compared with positive control (*p* < 0.05). However, there was no pronounced effect for 1/16 MIC and 1/8 MIC of rutin on biofilm formation of *S. suis* (*p* > 0.05) (**Figure 3B**).

Previous studies have shown that sub-MICs of antimicrobial agents can either increase or decrease biofilm formation by bacterial pathogens (Pompilio et al., 2010; Bedran et al., 2014). Our study brought clear evidence that sub-MICs of *Syringa oblata Lindl.* aqueous extract significantly decreased biofilm formation by *S. suis*. Previous studies have shown that extracts of *Syringa* plants mainly contain iridoids, lignans, and phenylethanoids that have antitumor, antihypertensive, anti-oxidant, and anti-inflammatory activities. We further provide evidence that rutin is the effective components of *Syringa oblata Lindl.* specifically affects *S. suis* biofilm. To the best of our knowledge, it was the first report that sub-MICs of *Syringa oblata Lindl.* aqueous extract decreased biofilm formed by *S. suis*.

### Direct Observation of Biofilm Formation *In vitro* by Scanning Electron Microscopy

**FIGURE 4:**
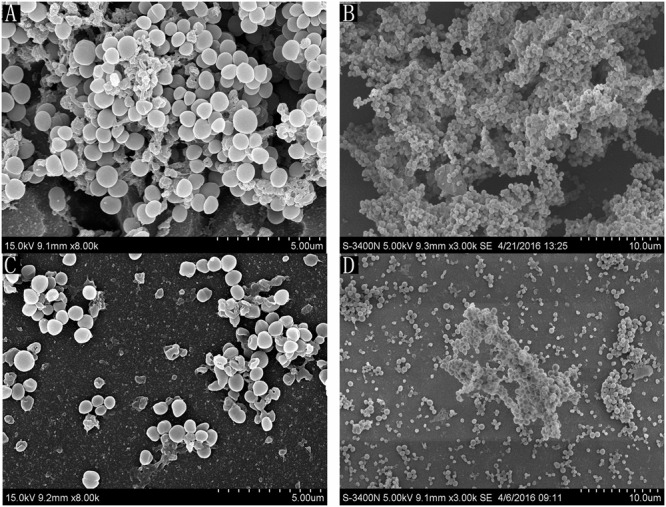
**Scanning electron micrographs of *S. suis* ATCC700794 biofilm following growth in THB supplemented without *Syringa oblata Lindl.* aqueous extract and rutin (A,C)**, or with 1/2 MIC of *Syringa oblata Lindl.* aqueous extract and rutin **(B,D)**. Controls refer to the absence of *Syringa oblata Lindl.* aqueous extract.

Scanning electron microscopy analysis was performed to observe the *Syringa oblata Lindl.* aqueous extract and rutin sub-MICs-induced *S. suis* biofilm formation. **Figures 4A,C** shows that the surface of the glass slide covered completely by a thick biofilm made of aggregates and microcolonies. However, when the culture medium was added with 1/2 MIC of *Syringa oblata Lindl.* aqueous extract and rutin, individual pairs of *S. suis* and individual short chains of *S. suis* attached to the glass slide (**Figures 4B,D**). Scanning electron microscopy analysis revealed that 1/2 MIC of *Syringa oblata Lindl.* aqueous extract and rutin significantly decreased biofilm formation.

### Differentially Expressed Proteins by iTRAQ

In the present study, iTRAQ technology was used to compare the patterns of protein expression at two different conditions (1/2 MIC of *Syringa oblata Lindl.* aqueous extract treated and non-treated cells). A ratio of proteins with >1.5 or <0.67 (*p*-value < 0.05) was considered to be differentially expressed. Based on this criterion, twenty-eight differentially expressed proteins were determined, 17 (61%) of which were up regulated and 11 (39%) were significantly suppressed (**Table 1**). The results regarding molecular function were as follows: motor activity (1, 3.6%), catalytic activity (17, 60.7%), DNA binding (5, 17.9%), metal ion binding (2, 7.1%), nucleotide binding (4, 14.3%), protein binding (1, 3.6%), and unknown molecular function (10, 35.7%). The results regarding cellular component were as follows: cytoplasm (4, 14.3%), membrane (7, 25.0%), cytoskeleton (1, 3.6%), chromosome (1, 3.6%), and unknown cellular component (18, 64.3%). According to the biological process, these proteins were classified into following categories: regulation of biological process (3, 10.7%), response to stimulus (3, 10.7%), metabolic process (12, 42.9%), cell organization and biogenesis (1, 3.6%), defense response (1, 3.6%), cellular homeostasis (1, 3.6%), transport (1, 3.6%), and unknown biological process (14, 50.0%). The majority of the proteins were related to catalytic activity and metabolism. A total of four proteins were classified as hypothetical, and their function is currently unknown. Additionally, three proteins were classified as “probable” and their likely identities were based upon homology to proteins of known function in other related species. For treated cells, various groups of related proteins were shown to be significantly up-regulated or down-regulated. These included those associated with metabolic process. These findings suggested that 1/2 MIC of *Syringa oblata Lindl.* aqueous extract treated cells and non-treated cells were subjected to different pharmic pressures, which might result in different proteome patterns.

**Table 1:** iTRAQ identification of differentially expressed proteins.

Accession	Proteins	Foldchange^a^
**Down-regulated proteins**		
G7S1J0	Phosphatase	0.618
G7RZ18	Sortase-like protein	0.644
B9WUV5	Transcriptional regulator, DeoR family	0.527
G7S2M0	DNA gyrase subunit B	0.624
D5AGH9	Antigen-like protein	0.624
G7SHZ3	Bacteriophage protein, putative	0.599
G5KZN4	DNA polymerase IV	0.573
M1VJJ3	CPS6J	0.585
M1VK55	CPS28H	0.568
G5L259	NADP-dependent glyceraldehyde-3-phosphate dehydrogenase, putative	0.581
G5KZR3	Glutathione *S*-transferase	0.658
**Up-regulated proteins**		
G5KZ86	Phosphatidylserine/phosphatidylglycerophosphate/cardiolipin synthase-like protein	1.766
J7KIA5	Abortive infection bacteriophage resistance related protein	1.527
	Putative competence-damage inducible protein	1.739
R4NU85	Phosphoribosylformylglycinamidine synthase domain-containing protein	1.964
G7SKQ9	CPS16F	2.981
E9NQ13	Putative uncharacterized protein	1.671
G5KZE4	Uncharacterized protein	1.568
M1VRG0	Putative uncharacterized protein	1.654
G7SP92	Putative uncharacterized protein	1.570
G5L3A6	Plasmid replication protein Rep and AAA-class ATPase domain protein	2.017
R4NW55	CPS16V	2.119
E9NQ29	Helicase	1.756
G7S7E3	Neprilysin (Fragment)	2.259
B0FYB8	ABC superfamily ATP binding cassette transporter, membrane protein	2.821
G7SD52	Type I site-specific restriction-modification system, R (Restriction) subunit and related helicase	2.538
G7SM99	Chloramphenicol acetyltransferase	2.862
C6GT52	FAD-dependent pyridine nucleotide-disulfide oxidoreductase	3.432
G7S7A9		

*^a^1/2 MIC of *Syringa oblata Lindl.* aqueous extract treated vs. non-treated cells.*

### Proteins Involved in Cell Growth and Metabolism

In the present study, *Syringa oblata Lindl.* aqueous extract affected metabolism, for example, DNA gyrase subunit B (foldchange: 0.62), DNA polymerase IV (fold change: 0.57), NADP-dependent glyceraldehyde-3-phosphate dehydrogenase (fold change: 0.58), Phosphatase (fold change: 0.62). The four proteins were related to cell growth and metabolism. Specifically, bacterial DNA gyrase is the target of many antibiotics (Oblak et al., 2007), such as fluoroquinolones.

### Proteins Involved in Capsule Expression

*Streptococcus suis* is a Gram-positive bacterium, and it possesses the CPS. Most *S. suis* strains possess a group of CPS synthesis genes and phenotypically express CPS. Previous studies have also shown that an *S. suis* serotype 2 mutant impaired in capsule expression acquire a biofilm-positive phenotype (Tanabe et al., 2010). Moreover, a hydrophilic capsule may hinder hydrophobic structures or components important for biofilm formation by *S. suis* (Bonifait et al., 2010). Alternatively, the infection is aggravated by unencapsulation by promoting the ability of bacterial cells to adhere to host cells and form thick biofilms (Benga et al., 2004; Bonifait et al., 2010; Tanabe et al., 2010; Lakkitjaroen et al., 2011). Many different surface molecules, including CPS, may play a fundamental role in *pneumococcal* biofilm development. Impaired *pneumococcal* CPS may increase biofilm formation (Qin et al., 2013). In this study, *Syringa oblata Lindl.* aqueous extract affected the CPS synthesis enzymes, for example, CPS 6J (fold change: 0.59), CPS 28H (fold change: 0.57), CPS 16F (fold change: 2.98), CPS 16V (fold change: 2.12). We speculated that *Syringa oblata Lindl.* aqueous extract might affect the biosynthesis of the CPS. However, further higher studies are needed to know the detailed molecular mechanism.

In summary, *Syringa oblata Lindl.* aqueous extract might be used as a potential inhibitor for the control of biofilm formation by *S. suis*. We found that *Syringa oblata Lindl.* aqueous extract affected growth, metabolism and the CPS synthesis enzymes. The active ingredient of the *Syringa oblata Lindl.* aqueous extract was rutin. These data showed a useful starting point for more focused studies to understand what exactly was going on. In future, the detailed molecular mechanism may provide further insight in this study.

## Author Contributions

JB the design whole experiment. YL directed the completion of the experiment. YY, SW, LG, JC, YR, WD, IM provided help during the experiment.

## Conflict of Interest Statement

The authors declare that the research was conducted in the absence of any commercial or financial relationships that could be construed as a potential conflict of interest.
